# Potential antigenic targets used in immunological tests for diagnosis of tegumentary leishmaniasis: A systematic review

**DOI:** 10.1371/journal.pone.0251956

**Published:** 2021-05-27

**Authors:** Mariana Lourenço Freire, Felipe Dutra Rêgo, Gláucia Cota, Marcelo Antônio Pascoal-Xavier, Edward Oliveira

**Affiliations:** Instituto René Rachou, Fundação Oswaldo Cruz, Belo Horizonte, Minas Gerais, Brazil; Universidade Federal da Bahia, BRAZIL

## Abstract

Immunological tests may represent valuable tools for the diagnosis of human tegumentary leishmaniasis (TL) due to their simple execution, less invasive nature and potential use as a point-of-care test. Indeed, several antigenic targets have been used with the aim of improving the restricted scenario for TL-diagnosis. We performed a worldwide systematic review to identify antigenic targets that have been evaluated for the main clinical forms of TL, such as cutaneous (CL) and mucosal (ML) leishmaniasis. Included were original studies evaluating the sensitivity and specificity of immunological tests for human-TL, CL and/or ML diagnosis using purified or recombinant proteins, synthetic peptides or polyclonal or monoclonal antibodies to detect *Leishmania*-specific antibodies or antigens. The review methodology followed PRISMA guidelines and all selected studies were evaluated in accordance with QUADAS-2. Thirty-eight original studies from four databases fulfilled the selection criteria. A total of 79 antigens were evaluated for the detection of antibodies as a diagnostic for TL, CL and/or ML by ELISA. Furthermore, three antibodies were evaluated for the detection of antigen by immunochromatographic test (ICT) and immunohistochemistry (IHC) for CL-diagnosis. Several antigenic targets showed 100% of sensitivity and specificity, suggesting potential use for TL-diagnosis in its different clinical manifestations. However, a high number of proof-of-concept studies reinforce the need for further analysis aimed at verifying true diagnostic accuracy in clinical practice.

## Introduction

Tegumentary Leishmaniasis (TL) is a neglected tropical disease caused by different species of the genus *Leishmania* (Kinetoplastea: Trypanosomatidae), transmitted to vertebrate hosts by sand flies (Diptera: Psychodidae) [1]. TL is considered an emergent and re-emergent disease, since a worrisome increase in its incidence has been reported [1]. On a the global scale, the number of new autochthonous TL cases reported annually to the World Health Organization (WHO) increased from 71,486 to 251,553 during 1998 to 2018 [2]. Several factors are involved with the spread of TL, such as human migration from rural to urban areas, conflicts and wars, disturbances in microenvironments due to climate change and human intervention and deterioration of socioeconomic conditions in endemic countries [3].

TL comprises a broad spectrum of clinical manifestations ranging from single or multiple ulcerative skin lesions (cutaneous leishmaniasis—CL), to diffuse (diffuse leishmaniasis-DL) and mucosal (mucosal leishmaniasis—ML) lesions, with the last two being typical in the Americas. TL is associated with physical deformities and psychological alterations, affecting the health and wellness of the patient [4, 5].

The range of clinical manifestations can hinder rapid and accurate diagnoses, a key step to initiate treatment promptly and control the disease. Although several advances, TL-diagnosis remains based on the triad of epidemiological background, clinical signs and laboratory diagnosis, including direct and histopathological examination of skin biopsy and molecular detection of *Leishmania* DNA. Despite high specificity, low sensitivities have been described for direct and histopathological examination, especially in New World countries, where chronic cases and ML are frequent [6–9]. Molecular techniques are complex, expensive, still without a standardized protocol for routine use and are restricted to reference and research centers. Therefore, these limitations make the TL-diagnosis scenario restricted, particularly in resource limited settings [10–12].

In this sense, immunological tests may present remarkable advantages for TL-diagnosis, due to the use of less invasive sampling compared to skin biopsy and their potential to be automated, quantitative and used as point-of-care tests. The anti-*Leishmania* delayed-type hypersensitivity reaction, known as the Montenegro skin test (MST), has been the most used immunological test for CL-diagnosis in Brazil, even though it presents significant limitations such as positive results associated with previous leishmaniasis or asymptomatic infections [13, 14]. Nonetheless, the production of the MST antigen was discontinued in Brazil, hampering even more CL-diagnosis in the country [15]. Other immunological tests, mainly Enzyme-Linked Immunosorbent Assay (ELISA), have presenting promising results in the Americas and beyond [7].

Several studies using soluble *Leishmania* antigen (SLA) in ELISA for TL-diagnosis, have presented variable sensitivity especially due to antigen preparation and antigenic differences among *Leishmania* isolates and species. Moreover, reduced specificity due to the cross-reactivity with other infectious diseases has been frequently reported [16–18]. Since CL-patients commonly produce low levels of anti-*Leishmania* antibodies, there is growing interest in high sensitivity antigens for immunological tests. Different methodologies have been employed, such as bioinformatics tools [19–23], cDNA expression library [24], phage display [25, 26], immunoproteomic approach [18, 27–32] and isolation and purification of glycoconjugates [33, 34] to identify potential antigens. Furthermore, immunological tools have already been used to detect *Leishmania* antigens using monoclonal and polyclonal antibodies by immunochromatographic test (ICT) or immunohistochemistry (IHC), such as the CL Detect Rapid Test (InBios International Inc., Seattle, WA, USA), which detects peroxidoxin from *Leishmania* and has been used especially in Old World countries, with limited sensitivity [35, 36].

In this sense, we consider immunnodiagosis as potential tools to increase the access and improve TL-diagnosis. Although systematic reviews have been conducted on some aspects of this form of diagnosis, it is essential to identify potential antigenic targets that have been evaluated as TL-immunodiagnostic, point out knowledge gaps that still remain and encourage other studies to allow its application in clinical practice [37, 38]. In this way, we performed a worldwide systematic review to identify potential antigenic targets, with reported sensitivity and specificity, used as TL-immunodiagnostic.

## Material and methods

### Protocol and registration

The review protocol was registered in the International Prospective Record of Systematic Reviews (PROSPERO: CRD42020213311) and was developed based on the Cochrane Handbook for Systematic Reviews of Diagnostic Test Accuracy [39]. This review followed the Preferred Reporting Items for Systematic Reviews and Meta-Analyses (PRISMA) (S1 Table) [40].

### Information sources and study selection

Structured searches were conducted in the following databases: MEDLINE, Virtual Health Library, Embase and Cochrane. A comprehensive list of key terms including tegumentary leishmaniasis and its different clinical forms AND immunological diagnosis or targets (antigens and antibodies) AND techniques or outcomes (sensitivity and specificity), was constructed in MEDLINE (S1 Fig). Similar searches were adapted to each database. Complementary searches were performed by analysis of reference lists of selected articles. Searches were performed on 23^rd^ March 2020, without restriction of publication date.

### Inclusion and exclusion criteria

Original research articles reporting on the performance (sensitivity and specificity) of immunological tests based on the detection of antibodies or antigens using purified or recombinant proteins, synthetic peptides or polyclonal or monoclonal antibodies for diagnosis of human-TL, CL or ML were included. Exclusion criteria were: evaluation of serological tests based on SLA; only non-human samples were tested (e.g. canine samples); both sensitivity and specificity of the immunological tests were not presented or were impossible to be calculated; less than five samples were tested; the absence of information about the reference test and a non-specific *Leishmania* antigen was used.

### Selection process

For each database, all publications were retrieved and duplicate citations were excluded by EndNote software [41]. Based on the inclusion and exclusion criteria, two independent reviewers analyzed each publication by title and abstract using Rayyan software [42]. Articles with no reason for rejection were included for full text reading. All discrepancies were solved by consensus after discussion. Selected studies were read in full to confirm their eligibility, to extract data or to exclude if exclusion criteria were identified during this step.

### Data extraction

Data were independently extracted by two researchers (MLF and FDR) directly from full-length articles and were checked by a third researcher (EO). In case of disagreements, the final decision was reached by consensus. In this study, data were extracted and a 2x2 contingency table set up for immunological tests, containing the true positives, false positives, true negatives and false negatives. Furthermore, the following items were extracted: origin of the participants; the immunological test used; antigen or antibody types; *Leishmania* species and reference standard test used for disease confirmation. The phase of development of each study was classified according to Leeflang & Allerberger (2019) [43].

### Study quality assessment

The quality of the studies was assessed using the second version of Quality Assessment of Studies of Diagnostic Accuracy Approach (QUADAS-2) [44]. This tool allows a more transparent rating of risk of bias for studies included in systematic reviews on diagnostic accuracy.

### Data synthesis

The performance of antigenic targets was presented in four groups according immunological tests and clinical form: 1) ELISA for TL; 2) ELISA for CL; 3) Other immunological tests for CL and 4) ELISA for ML. The performance outcomes for each antigen or antibody were sensitivity (probability of a positive test among cases or disease confirmed individuals) and specificity (probability of a negative test among controls or individuals without disease). Forest plots showing sensitivity and specificity values of all antigens, including 95% confidence intervals (CI) and Summary Receiver Operating Characteristic (SROC) curves were created using RevMan 5.3.

Several studies considered a set of results for the same antigen (e.g. different cut-off points were available or different non-case groups were used in the analysis, such as healthy patients and those with other diseases). If possible, these results were grouped and only one sensitivity rate and one specificity rate including all evaluated patients. When impossible, we chose to present data that reflect the best field conditions (e.g. non-case group of patients with other diseases) or the better performance (e.g. cut-off point with best performance).

## Results

### Literature search

**Fig 1:**
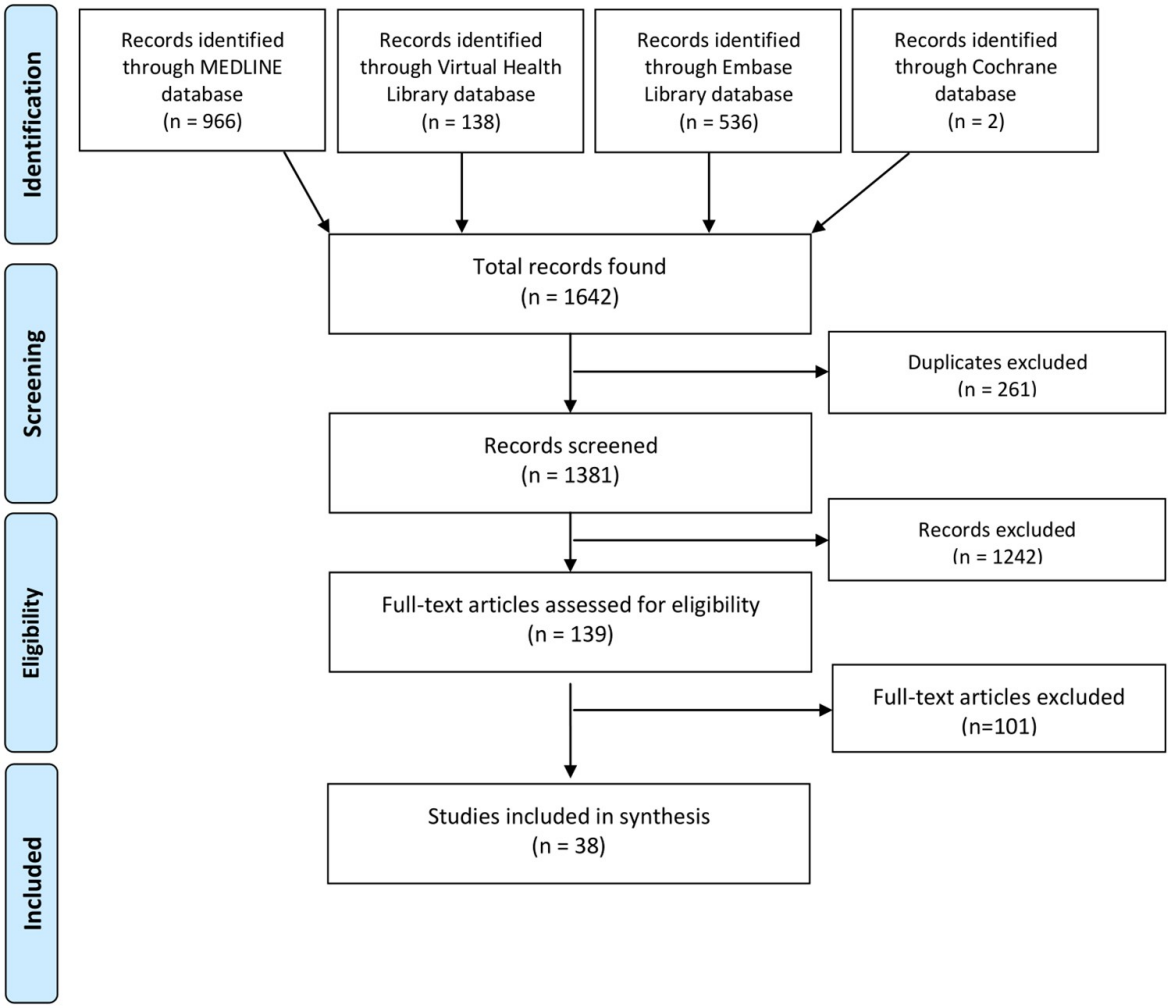
Flow diagram illustrating the study selection process according to PRISMA.

A total of 1642 articles from four databases were initially identified. Of this total, 261 were excluded due to duplicity (the same study was found in different databases). The title and abstract of each of the 1381 articles were checked and 139 were selected for full text reading. Finally, 98 articles presented exclusion criteria and so 38 were included (Fig 1).

### Descriptive analysis of included studies

The characteristics of all included studies are presented in Table 1. In several studies, test performance was analyzed according to the clinical form (CL and ML) or globally (TL). In 19 studies, the antigenic targets were evaluated for TL-diagnosis, in 21 for CL and in 9 for ML. Sample size ranged from 26 to 500 patients. A total of three different immunological tests using purified or recombinant proteins, synthetic peptides or polyclonal or monoclonal antibodies were reported: ELISA, ICT and IHC. Different reference standard tests were used to confirm leishmaniasis cases. Thirty-one studies (81.6%) considered at least one parasitological method as a reference standard test, such as microscopy examination or *in vitro* culture for isolation of the parasite. On the other hand, seven studies (18.4%) considered some immunological or molecular tests as a reference standard. A total of 89.5% (34 out of 38) of the studies was classified as phase I (proof-of-concept), and the remaining 10.5% (4 out of 38) was classified as phase III.

**Table 1:** Characteristics of the studies included in the systematic review.

Reference	Country	Case (n)	No-case (n)	Reference standard test	Test platform	Protein targets	Type	Clinical form evaluated	Phase
Bennis et al., 2018 [35]	Morocco	136	83	Microscopy and/or PCR	ICT	Peroxidoxin	pAb	CL	III
Carmelo et al., 2002 [45]	Peru	24	44	Microscopy and culture	ELISA	H1 and 7 peptides	RP / SP	CL	I
Carvalho et al., 2017 [29]	Brazil	57	55	Microscopy and PCR	ELISA	HP (XP_001469551.1)	RP	TL	I
Celeste et al., 2004 [46]	Brazil	26	20	MST and/or hitopathology and IFAT	ELISA	HSP83	RP	CL and ML	I
Celeste et al., 2014 [47]	Brazil	26	109	Microscopy and Immunological	ELISA	HSP83	RP	CL and ML	I
Coelho et al., 2016 [30]	Brazil	24	28	Microscopy and PCR	ELISA	Cytochrome c oxidase and Putative IgE histamine releasing factor	RP	TL	I
Costa et al., 2016 [25]	Brazil	50	10	Microscopy and PCR	ELISA	A10, C11, C12 B10, B7 and H7	SP	TL	I
de Silva et al., 2017 [48]	Sri Lanka	59	22	PCR	ICT	Peroxidoxin	pAb	CL	III
Duarte et al., 2015 [27]	Brazil	43	40	Microscopy, PCR and MST	ELISA	Enolase; eukaryotic initiation factor 5a; HP (LbrM.30.3350); tryparedoxin peroxidase and β-tubulin	RP	TL	I
Gomes-Silva et al., 2008 [33]	Brazil	58	171	Microscopy and immunological	ELISA	Con-A and Jaca bound fraction	PP	TL	I
González et al., 2002 [49]	Peru	20	19	Microscopy and culture	ELISA	23085, 23089 and 23083	SP	TL	I
Jensen et al. 1996 [50]	Sudan	33	88	Microscopy and histopathology	ELISA	GPB and Gp63	SP / PP	CL	I
Kenner et al., 1999 [51]	Central America	41	20	Culture	IHC	G2D10	mAb	CL	I
Lage et al., 2019 [18]	Brazil	50	75	Microscopy and PCR	ELISA	A2 and HP (XP_003886492.1)	RP	TL	I
Lima et al., 2017 [52]	Brazil	45	50	Microscopy, PCR and MST	ELISA	HP (XP_001566959.1)	RP	TL, CL and ML	I
Lima et al., 2018 [31]	Brazil	40	143	Microscopy and PCR	ELISA	Enolase; eukaryotic initiation factor 5a; HP (XP_001566959.1) and β-tubulin	RP	TL	I
Link et al., 2017 [26]	Brazil	57	30	ELISA	ELISA	P1 and MIX (P1 + P2 + P3)	SP	CL	I
Longoni et al., 2014 [53]	Colombia	51	10	Microscopy	ELISA	Fe-SOD	PP	CL	I
Marin et al., 2009 [54]	Spain	113	32	Microscopy	ELISA	Fe-SOD	PP	CL and ML	I
Menezes-Souza et al., 2014a [19]	Brazil	65	70	Microscopy and PCR	ELISA	Peroxidoxin	RP	TL, CL and ML	I
Menezes-Souza et al., 2014b [20]	Brazil	65	70	Microscopy and PCR	ELISA	HSP83 and 3 peptides	RP/SP	TL, CL and ML	I
Menezes-Souza et al., 2015a [21]	Brazil	65	70	Microscopy and PCR	ELISA	Cathepsin L-like and peptide	RP / SP	TL	I
Menezes-Souza et al., 2015b [22]	Brazil	65	70	Microscopy and PCR	ELISA	MAPK3 and MAPK4	RP	TL	I
Montoya et al., 1997 [55]	Colombia/Peru	78	39	Serologic	ELISA	T26-U2 and T26-U4	RP	TL	I
Padilla et al., 2003 [56]	Peru	18	8	ELISA	ELISA	Acidic ribosomal P2β proteins	RP	TL	I
Salles et al., 2019 [32]	Brazil	40	100	Microscopy and PCR	ELISA	Small myristoylated protein-3 and peptide	RP/SP	TL	I
Sato et al., 2017 [57]	Brazil	219	281	Microscopy, PCR and/or histopathology	ELISA	LB6H e Lb8E	RP	TL	I
Schallig et al., 2019 [58]	Suriname	79	14	Microscopy or PCR	ICT	Peroxidoxin	pAb	CL	III
Shirian et al., 2014 [59]	Iran	100	30	Cytology or histology and PCR	IHC	IS2-2B4 (A11) and XLVI-5B8- B3 (T1)	mAb	CL	I
Skraba et al., 2014 [60]	Brazil	60	177	Microscopy	ELISA	Mix (36 and 48–56 kDa)	PP	CL	I
Soto et al., 1996 [61]	Spain	21	30	Microscopy and IFAT	ELISA	Acidic ribosomal protein family (LiP2a-Q and LiP2b-Q)	RP	ML	I
Souza et al., 2013 [62]	Brazil	102	180	MST, immunologic, histopathology and/or therapeutic test	ELISA	H2A; H2B; H3; H4; HSP70; KMP11	RP	TL, CL and ML	I
de Souza et al., 2018 [34]	Brazil	30	119	Microscopy and culture	ELISA	NGP 0204; NGP2333; NGP 2334; NGP 2203	PP	CL	I
de Souza et al., 2019 [63]	Brazil	74	63	Microscopy	ELISA	Lbk39	RP	CL	I
Vidigal et al., 2008 [64]	Brazil	48	114	Microscopy, MST and IFAT	ELISA	Fraction 8—peak 2	PP	CL	I
Vink et al., 2018 [36]	Afghanistan	257	17	Microscopy and/or PCR	ICT	Peroxidoxin	pAb	CL	III
Yeganeg et al., 2009 [65]	Iran	30	41	Microscopy	ELISA	Fe SOD-B1	RP	CL	I
Zurita et al., 2003 [66]	Peru	50	36	Culture	ELISA	HSP70 and 5 peptides	RP	TL, CL and ML	I

CL—cutaneous leishmaniasis; ML—mucosal leishmaniasis; TL—tegumentary leishmaniasis; HP—hypothetical protein; RP—recombinant protein; PP—purified protein; SP—synthetic peptide; mAb—monoclonal antibody; pAb—polyclonal antibody.

### ELISA for TL diagnosis

**Fig 2:**
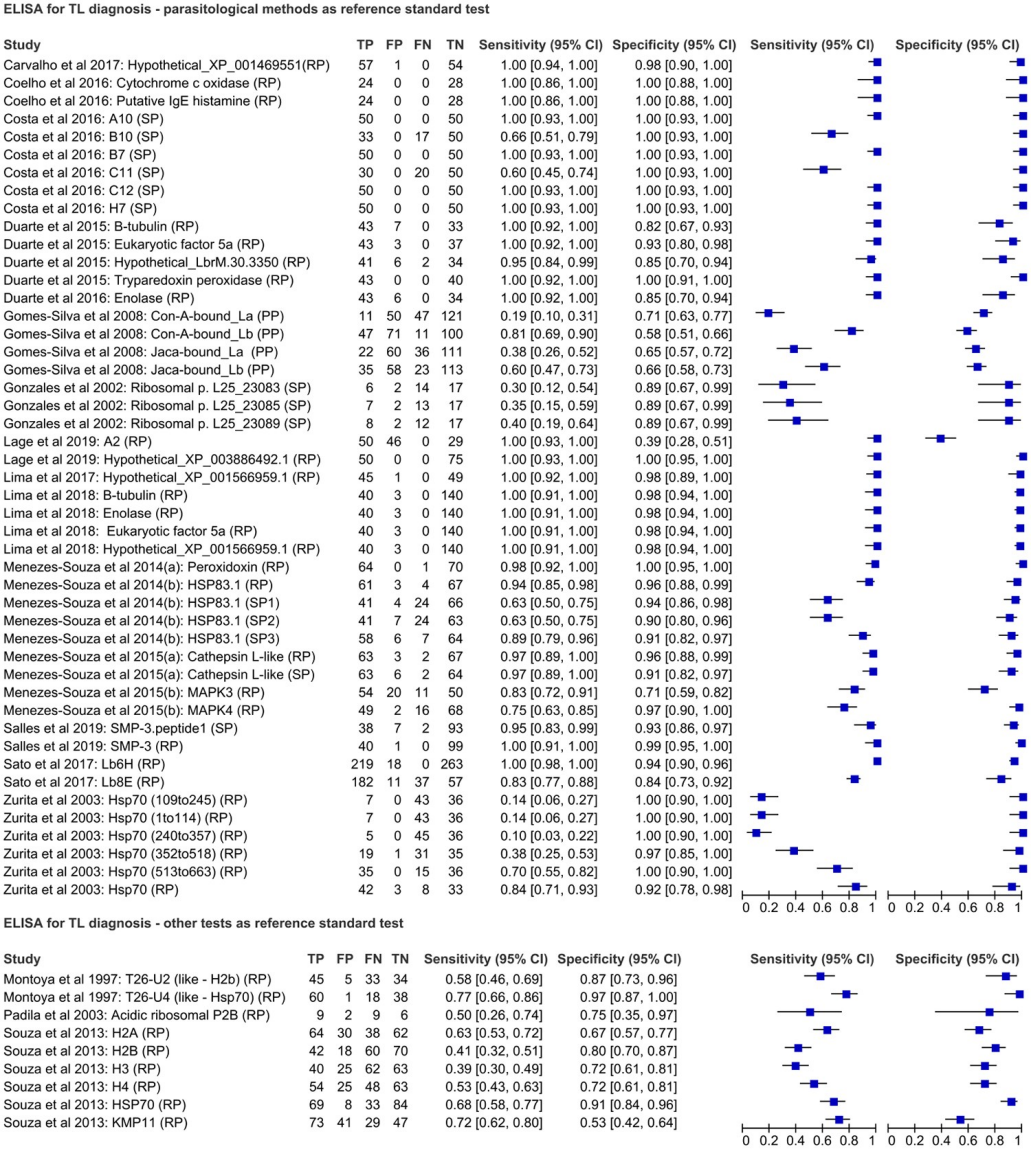
Forest plot representing sensitivity and specificity indices of ELISA using different antigenic targets for TL diagnosis.

Nineteen studies used ELISA to evaluate the performance of a total of 56 antigens for TL-diagnosis, without specification of the clinical form (CL or ML). These studies evaluated 38 recombinant proteins, 14 synthetic peptides and 4 purified proteins. Forty-seven antigens were evaluated in studies that considered at least one parasitological method, such as microscopy examination or *in vitro* culture isolation of the parasite, as a reference standard test. The number of TL-patients ranged from 20 to 219 and the number of non-TL patients ranged from 8 to 281. The highest performance (100% of sensitivity and specificity) was reported for four recombinant proteins (cytochrome c oxidase; hypothetical protein XP_003886492.1; putative IgE histamine releasing factor; tryparedoxin peroxidase) and four synthetic peptides (A10, B7, C12 and H7) selected by the phage display technique [18, 25, 27, 30]. Nine other antigens were evaluated in studies that considered at least one immunological method as a reference standard test. For these antigens the sensitivity ranged from 39.8% to 76.9% and the specificity from 53.4% to 97%. The forest plots for sensitivity and specificity of ELISA considering parasitological methods and other tests as reference standard tests for TL-diagnosis are presented in Fig 2; more details about each evaluated antigen are available in S2 Table.

### ELISA for CL diagnosis

**Fig 3:**
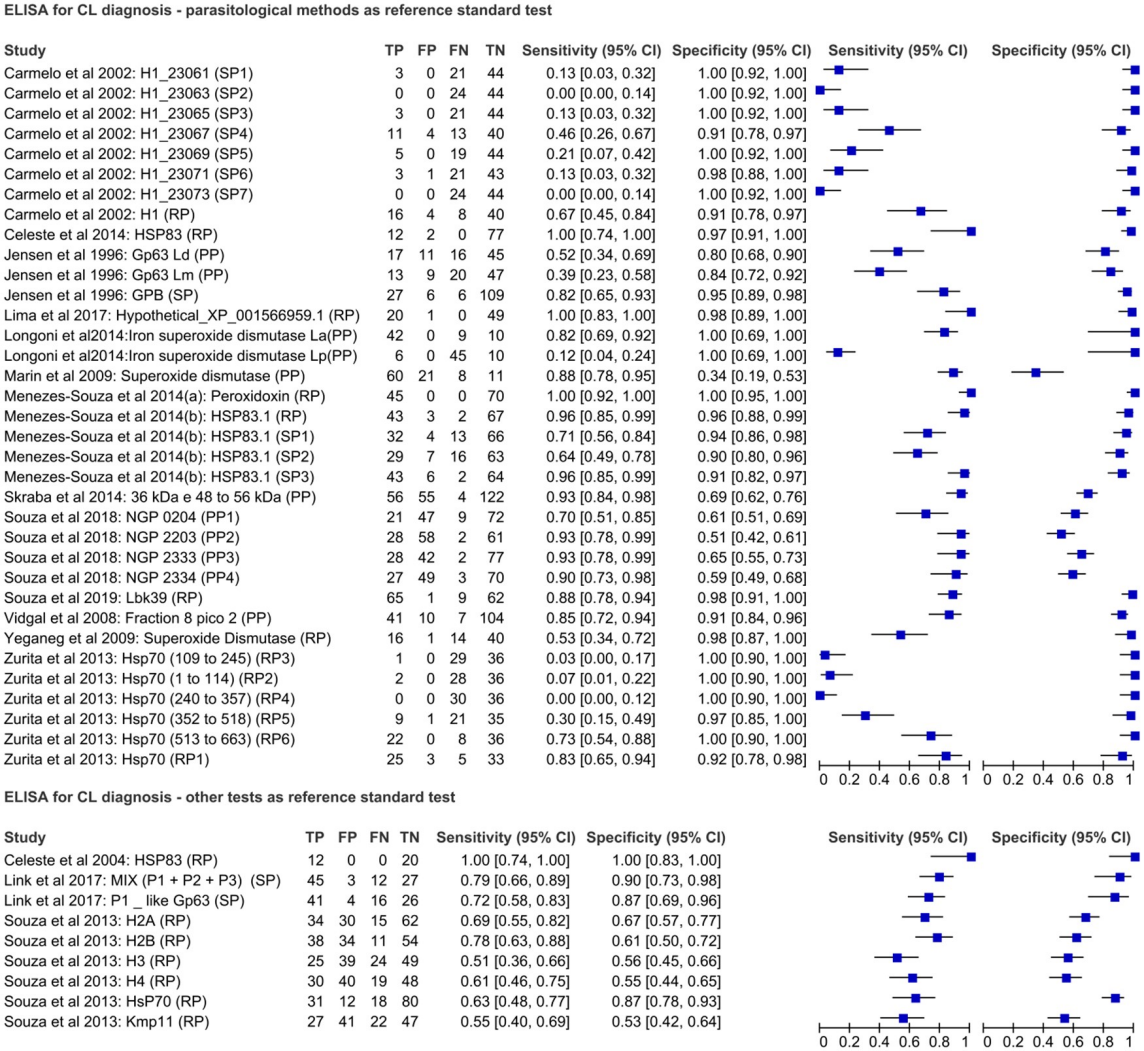
Forest plot representing sensitivity and specificity indices of ELISA using different antigenic targets for CL-diagnosis.

Seventeen studies used ELISA to evaluate the performance of 44 antigens for CL-diagnosis, which comprised 20 recombinant proteins, 13 synthetic peptides and 11 purified proteins. The performance of 35 antigens was evaluated considering at least one parasitological method as a reference standard test. Among these, the sample size for studies of CL-patients ranged from 12 to 74 and for non-CL-patients from 10 to 177. Peroxidoxin was the only antigen presenting 100% sensitivity and specificity [19]. Nine antigens were evaluated considering at least one immunological test as a reference standard. Overall, HSP83 presented the highest performance (100% sensitivity and specificity) [46] (Fig 3, S3 Table).

### Other immunological tests for CL diagnosis

**Fig 4:**
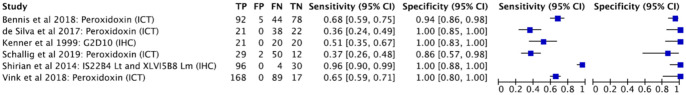
Forest plot representing sensitivity and specificity indices of other immunological tests using different antigenic targets for CL-diagnosis.

The performance of ICT and IHC using different monoclonal and/or polyclonal antibodies is presented in Fig 4 and detailed in S4 Table. Four studies evaluated the CL Detect Rapid Test (InBios International Inc., Seattle, WA, USA) in different countries. The sensitivity ranged from 35.6 to 67.6 and the specificity was higher than 80%. For IHC, two monoclonal antibodies were employed to detect antigens in fixed skin fragments. The highest performance was reported for IS22B4/XLVI5B8 mAbs, with 96% and 100% sensitivity and specificity, respectively [59].

### ELISA for ML results

**Fig 5:**
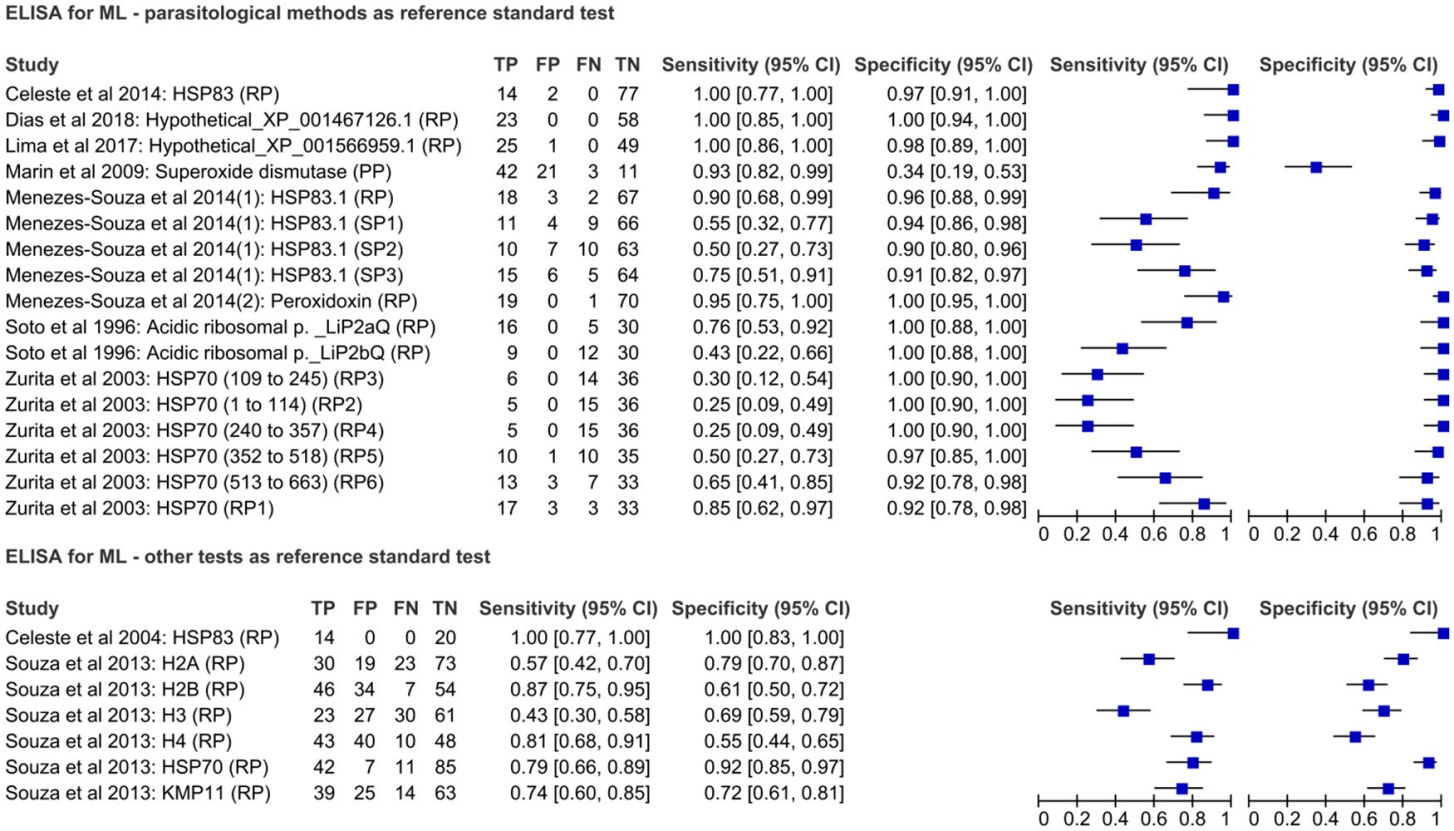
Forest plot representing sensitivity and specificity indexes of other immunological tests using different antigenic targets for ML-diagnosis.

Nine studies used ELISA to evaluate the performance of 23 antigens for ML-diagnosis, which comprised 19 recombinant proteins, three synthetic peptides and one purified protein. The sample size from ML-patients in these studies ranged from 14 to 53 and from non-ML-patients from 20 to 92. At least one parasitological method was used as a reference standard test for the evaluation of sixteen antigens. The highest performance was obtained for Hypothetical protein XP_001467126.1, with 100% sensitivity and 98% specificity [27]. Seven antigens were evaluated in studies considering at least one immunological test as a reference standard. As noted for CL-diagnosis, 100% sensitivity and specificity were reported for HSP83 [46]. The performance of these antigens is presented in Fig 5 and more details are available in S5 Table.

**Fig 6:**
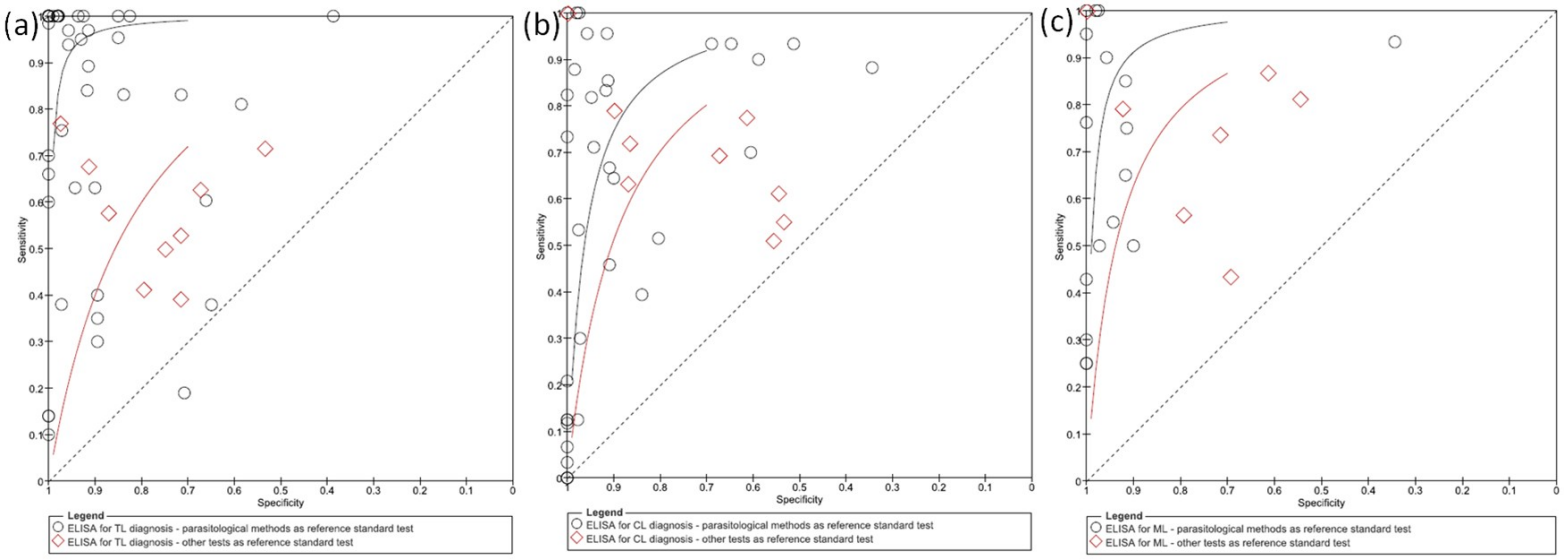
SROC curve for diagnosis of TL (a), CL (b) and ML (c) according to reference standard test.

The SROC curves with the antigen performances for the diagnostic of different clinical forms, using parasitological or other tests (such as ELISA and MST) as a reference standard, are presented in Fig 6. The antigens tended to have greater accuracy in studies that have used the parasitological methods as reference standard tests, regardless of TL-clinical manifestation.

### Quadas-2 based quality assessment

**Fig 7:**
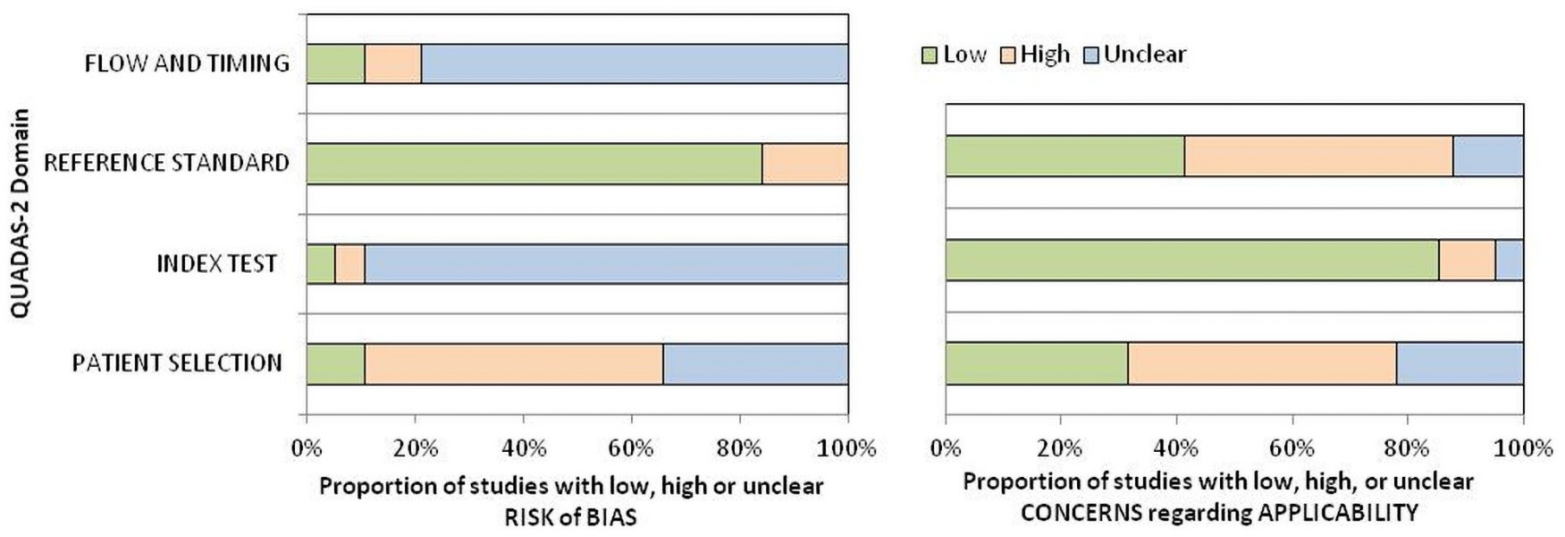
Risk of bias assessed by the QUADAS-2 tool according to different study characteristics (patient selection, index test, reference standard and flow and timing).

Quality assessment of the study according to risk of bias and concern with applicability (low, high and unclear) is shown in Fig 7. Of the 38 studies assessed, 21 had high risk of bias in patient selection. The risk was unclear for the index test in 34 studies and for flow and timing in 30 studies. Nineteen studies had high concerns regarding applicability of patient selection criteria.

## Discussion

TL is considered a multifactorial disease, responsible for psychological and social impacts due to scars and mutilating lesions generating stigma and self-deprecation in affected patients [67]. Improvements in healthcare access and laboratory diagnosis are needed to overcome the impacts of this disease and should be encouraged [68]. According to WHO’s Special Programme for Research and Training in Tropical Diseases (TDR), the ideal test must be affordable, sensitive, specific, user-friendly, rapid, equipment-free and delivered to end-users (ASSURED) [69]. Immunological tests may fill these criteria since they are usually easy to perform, accessible and require minimally invasive sample collection. Therefore, the identification of sensitive and specific antigenic targets seems to be a promising step toward the improvement of TL-diagnosis.

The studies analyzed here were conducted from 1996 to 2019, however, almost 50% of them were conducted in the last five years, mostly in Brazil or another country in the Americas. The increase in the number of studies is coincident with the interruption of the production of MST antigen in Brazil in 2015, which extinguished the simple and rapid immunodiagnostic for TL [15]. This fact may have boosted research aimed at finding new diagnostic tools.

Parasitological diagnosis was considered a reference standard test in 89.5% of the studies. Despite this technique being highly specific for TL-diagnosis, its sensitivity is limited and inversely correlated with disease duration [9, 70]. However, no test seems to present sufficiently high sensitivity and specificity to be used as a gold standard test. We observed a tendency for index tests to be more accurate if parasitological tests were used as a reference standard than other reference test such as MST and histopathology (Fig 6). Polymerase chain reaction (PCR) was used as a reference standard test in 18 studies, generally in association with parasitological diagnosis. Overall, PCR appears to be a more suitable reference test, however, a standard protocol is urgently needed and encouraged, since distinct extraction methods, protocols and molecular targets have been used overtime [71].

The ability to accurately identify TL-patients is essential for a diagnostic test, in view of the range of clinical forms, disease severity and treatment toxicity. Several studies have included patients with Chagas disease as non-TL cases, however, despite phylogenetic proximity, the inclusion of patients with clinical signs that do not resemble TL is at least questionable. For tests with diagnostic purposes, a better sample panel needs to be encouraged, including diseases such as sporotrichosis, paracoccidioidomycosis, hanseniasis, vasculitis, syphilis and other dermal or mucosal diseases, that represent confounding factors in clinical practice.

Despite the distinct profiles in immune response usually reported for each clinical form of TL, some antigens presented high values of sensitivity, even for CL-patients. In general, higher levels of antibodies have been reported for ML-patients compared to CL-patients, the latter being characterized by a moderate Th1 immune response [72, 73]. In this way, it seems that problems related to antibody detection in CL-patients may be reduced by using sensitive targets and well-standardized procedures [16]. Some antigenic targets were evaluated for TL-diagnosis without distinction of clinical form and, consequently, immune response profile. We believe that the accuracy of these antigenic targets may be improperly estimated in these specific cases.

This systematic literature review found 79 different antigens, comprising 40 recombinant proteins, 24 synthetic peptides and 15 purified proteins. The identification and more refined selection of protein targets using recombinant proteins or synthetic peptides allows the development of more standardized techniques due to the possibility of generating the purest inputs. Some protein-families have been widely evaluated as antigenic targets for TL-immunodiagnosis, such as heat shock proteins (HSPs), histones and peroxiredoxins, with promising results.

HSPs represent a highly conserved family of intracellular proteins of varying molecular weights in prokaryotic and eukaryotic cells, including cytosolic, mitochondrial, nuclear and endoplasmic reticulum resident proteins. They act as a chaperon in peptide folding and in the translocation of proteins to organelles, the prevention of protein aggregation, and the stabilization and degradation of proteins [74, 75]. HSPs have usually been identified by amino acid sequence homology and molecular weight, with HSP70 and HSP83 being the most abundant [76, 77]. These proteins are constitutively expressed throughout the life cycle of *Leishmania*, increasing expression in the vertebrate host due to variation in temperature and pH [78]. The recombinant proteins HSP70 and HSP83, and the synthetic peptides extracted from those proteins, have been widely evaluated for TL-diagnosis [20, 24, 46, 47, 62, 66]. The performance of these targets seems to be promising, with HSP83 presenting sensitivity of over 90% and high specificity with few cross reactions [20, 46, 47].

Histones are conserved proteins bound to DNA establishing chromatin structure in eukaryotes. Several biological functions have been described for histones during *Leishmania* infection in susceptible hosts. Core nucleosomal *Leishmania* histones have been proposed as prominent intracellular pathoantigens, since immunological responses against histones seem to be involved in the pathological mechanisms of visceral leishmaniasis (VL) [79, 80]. In this way, this protein family has been extensively employed in ELISA for both human and canine VL [81–84]. The presence of antibodies against rH2B of *L*. *peruviana* [55], rH1 of *L*. *braziliensis* [45] and rH2A, rH2B, rH3 and rH4 of *L*. *infantum* have been detected in sera from CL or ML patients. CARMELO et al. (2002) demonstrated that the antibody against histone H1 was specific for the parasite without cross reaction with human histones. However, moderate cross reactivity has been observed in a sample panel composed of Systemic Lupus Erythematosus (SLE) and Chagas disease [45, 55, 62].

Peroxidoxin, also known as thiol-specific antioxidant protein, as well as tryparedoxin peroxidase, are peroxiredoxins, an antioxidant enzyme family [85–87]. This protein family has been described in a wide variety of organisms and several biological functions have been reported for *Leishmania* parasites, such as virulence factor and protection against reactive oxygen and nitrogen species [88]. In this manner, they are directly associated with cell proliferation, senescence, apoptosis, and circadian rhythms [89]. These proteins have been described in the secretome of *L*. *braziliensis* and the antigenicity of tryparedoxin peroxidase has also been evaluated for both human and canine VL-diagnosis [90–92]. Peroxidoxin is the protein target identified by the CL Detect Rapid Test (InBios International Inc.) for CL-diagnosis. Variable performance has been reported for this ICT, according to endemic region and, consequently, the *Leishmania* species involved, with better results for infections caused by *L*. *tropica*, with sensitivity ranging 65.4–73% and specificity 92–100% [35, 36]. This test, however, has not been evaluated in Brazil.

Other recombinant proteins, such as cytochrome c oxidase, putative IgE histamine releasing factor, prohibitin, eukaryotic initiation factor 5a, cathepsin L-like peptide and small myristoylated protein-3, as well as hypothetical proteins, were evaluated in preliminary studies demonstrating potential as candidates for TL-immunodiagnosis, and so more studies are desirable [27, 29, 30, 93].

Some promising synthetic peptides have been identified and employed in ELISA. The use of small fragments containing potent antigenic determinants is able to minimize non-specific reactions. LINK et al. (2017) identified three peptides by phage display, probably from GP63 glycoprotein, and presented 79% sensitivity in ELISA [26]. COSTA et al. (2016) found high performance for three clones (A10, C12 and H7) in discriminating TL-patients from patients with other diseases and healthy individuals (100% sensitivity and specificity) [25]. However, these short linear peptides may have some drawbacks, such as limited passive adsorption on polystyrene titration plates (ELISA-standard procedure), inability to identify serum antibodies that recognize conformational epitopes and problems considering reproducibility due to variation in inter-assay reactivity producing different batches [94].

Despite the advantages, the absence of post-translational modifications of bacterially-expressed and chemically synthesized proteins comprises an important limitation for the employment of this biotechnology for immunodiagnosis. In this way, purified proteins can represent significant advantages, especially regarding immunoreactivity. This review found iron-superoxide dismutase to be a purified protein with interesting results, with more than 80% sensitivity for CL or ML diagnosis. However, being purified proteins, sensitivity and specificity may vary according to the type, source, and purity of the antigen used [53, 54].

Three polyclonal and monoclonal antibodies were evaluated for detecting *Leishmania* antigen by ICT and IHC [35, 36, 48, 51, 58, 59]. The phase III studies included were ICT tests, that is, prospective studies in which the index and reference test were performed simultaneously in patients with clinical suspicion [35, 36, 48, 58]. This is a commercial test that, despite its low sensitivity, has been useful in some localities due to the simple realization and high specificity, reducing the number of CL patients referred for diagnosis confirmation. High performance was observed in phase I studies for species-specific monoclonal antibody (IS2-2B4—A11/ XLVI-5B8-B3) employed in IHC, with 96% sensitivity and 100% specificity [59]. More robust studies using monoclonal or polyclonal antibodies for TL-diagnosis need to be encouraged evaluating the performance in clinical practice.

The strength of the present literature review is that it employed a comprehensive search strategy with four databases. One of the meaningful limitations may be the limited number of studies evaluating the same protein target, and so results need to be interpreted with caution. For this reason, a meta-analysis was not performed here. Additionally, it is important to consider that the risk of bias for many of the included studies was unclear and/or was high for some of the evaluated parameters: “Patient selection”, “Flow and Timing” and "Index test". Here, we identified a large number of antigenic targets that could help clinical diagnosis. However, the high number of proof-of-concept and phase I studies highlights the need to move forward with more refined and mainly prospective studies including patients with clinical suspicion of TL from different endemic regions and the most sensitive reference standard tests, to evaluate the diagnostic accuracy of antigenic targets reported in clinical practice.

## Supporting information

S1 Fig Terms used in MEDLINE search. (TIF)

S1 Table PRISMA checklist. (DOCX)

S2 Table Antigenic targets used in ELISA for diagnosis of tegumentary leishmaniasis. (DOCX)

S3 Table Antigenic targets used in ELISA for diagnosis of cutaneous leishmaniasis. (DOCX)

S4 Table Antigenic targets for diagnosis of cutaneous leishmaniasis by other tests. (DOCX)

S5 Table Antigenic targets used in ELISA for diagnosis of mucosal leishmaniasis. (DOCX)
