# Supplementary figures and images for: Potential antigenic targets used in immunological tests for diagnosis of tegumentary leishmaniasis: A systematic review
Source: PLoS One. 2021 May 27;16(5):e0251956. doi: 10.1371/journal.pone.0251956 (PMC8158869; doi:10.1371/journal.pone.0251956)

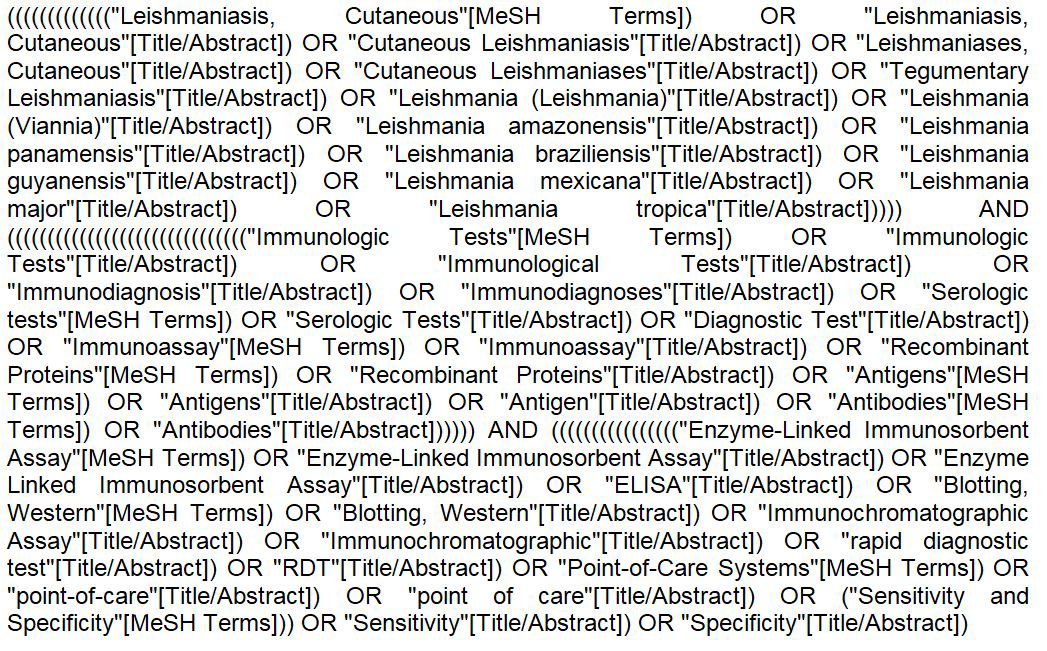

Supplement: S1 Fig — (TIF) [file pone.0251956.s001.tif]
