# Supplementary material for: Potential antigenic targets used in immunological tests for diagnosis of tegumentary leishmaniasis: A systematic review
Source: PLoS One. 2021 May 27;16(5):e0251956. doi: 10.1371/journal.pone.0251956 (PMC8158869; doi:10.1371/journal.pone.0251956)
Supplement: S2 Table — (DOCX) [file pone.0251956.s003.docx]

**S2 Table.** Antigenic targets used in ELISA for diagnosis of tegumentary leishmaniasis.

| **Antigen *(Leishmania* species)** | **Antigen Type** | **Origin of samples** | **Reference standard test** | **TL patients** | **Control (total)** | **Sensitivity (%)** | **Specificity (%)** | **Reference** |
| --- | --- | --- | --- | --- | --- | --- | --- | --- |
| A2 (Li) | RP | Brazil | Microscopy and PCR | TL=50 (CL=25/ML=25) | 75 (HC=25/DC=50) | 100.0 | 38.6 | Lage et al., 2019 |
| Acidic ribosomal P2β proteins (Lb) | RP | Peru | ELISA | TL=18 (CL=5/ML=13) | DC=8 | 50.0 | 75.0 | Padilla et al., 2003 |
| Cathepsin L-like (Lb)^#^ | RP | Brazil | Microscopy and PCR | TL=65 (CL=45/ML=20) | 70 (HC=50/DC=20) | 96.9 | 95.7 | Menezes-Souza et al., 2015 |
| Cathepsin L-like_peptide (Lb) ^#^ | SP | Brazil | Microscopy and PCR | TL=65 (CL=45/ML=20) | 70 (HC=50/DC=20) | 96.9 | 91.4 | Menezes-Souza et al., 2015 |
| Cytochrome c oxidase (Li) ^#^ | RP | Brazil | Microscopy and PCR | TL=24 (CL=12/ML=12) | 28 (HC=20/DC=8) | 100.0 | 100.0 | Coelho et al. 2016 |
| Enolase (Lb) | RP | Brazil | Microscopy, PCR and MST | TL=43 (CL=23/ML=20) | 40 (HC=30/DC=10) | 100.0 | 85.0 | Duarte et al., 2015 |
| Enolase (Lb) ^#^ | RP | Brazil | Microscopy and PCR | TL=40 (CL=15/ML=25) | 143 (HC=75/DC=68) | 100.0 | 97.8 | Lima et al., 2018 |
| Eukaryotic initiation factor 5a (Lb) ^#^ | RP | Brazil | Microscopy, PCR and MST | TL=43 (CL=23/ML=20) | 40 (HC=30/DC=10) | 100.0 | 92.5 | Duarte et al., 2015 |
| Eukaryotic initiation factor 5a (Lb) ^#^ | RP | Brazil | Microscopy and PCR | TL=40 (CL=15/ML=25) | 143 (HC=75/DC=68) | 100.0 | 97.8 | Lima et al., 2018 |
| H2A (Li) | RP | Brazil | MST, serologic, histopathology and/ or therapeutic test | TL=102 (CL=49/ML=53) | DC=92 | 62.9 | 67.0 | Souza et al., 2013 |
| H2B (Li) | RP | Brazil | MST, serologic, histopathology and/ or therapeutic test | TL=102 (CL=49/ML=53) | HC=88 | 41.2 | 79.5 | Souza et al., 2013 |
| H3 (Li) | RP | Brazil | MST, serologic, histopathology and/ or therapeutic test | TL=102 (CL=49/ML=53) | HC=88 | 39.8 | 71.5 | Souza et al., 2013 |
| H4 (Li) | RP | Brazil | MST, serologic, histopathology and/ or therapeutic test | TL=102 (CL=49/ML=53) | HC=88 | 52.5 | 71.5 | Souza et al., 2013 |
| HSP70 (Li) | RP | Brazil | MST, serologic, histopathology and/ or therapeutic test | TL=102 (CL=49/ML=53) | DC=92 | 67.9 | 91.3 | Souza et al., 2013 |
| HSP70 (Lb) | RP | Peru | Culture | TL=50 (CL=30/ML=20) | 36 (HC=20/DC=16) | 84.0 | 91.7 | Zurita et al., 2003 |
| HSP70 (1–114) (Lb) | RP | Peru | Culture | TL=50 (CL=30/ML=20) | 36 (HC=20/DC=16) | 14.0 | 100.0 | Zurita et al., 2003 |
| HSP70 (109–245) (Lb) | RP | Peru | Culture | TL=50 (CL=30/ML=20) | 36 (HC=20/DC=16) | 14.0 | 100.0 | Zurita et al., 2003 |
| HSP70 (240–357) (Lb) | RP | Peru | Culture | TL=50 (CL=30/ML=20) | 36 (HC=20/DC=16) | 10.0 | 100.0 | Zurita et al., 2003 |
| HSP70 (352–518) (Lb) | RP | Peru | Culture | TL=50 (CL=30/ML=20) | 36 (HC=20/DC=16) | 38.0 | 97.2 | Zurita et al., 2003 |
| HSP70 (513–663) (Lb) | RP | Peru | Culture | TL=50 (CL=30/ML=20) | 36 (HC=20/DC=16) | 70.0 | 100.0 | Zurita et al., 2003 |
| HSP83.1 (Lb) ^#^ | RP | Brazil | Microscopy and PCR | TL=65 (CL=45/ML=20) | 70 (HC=50/DC=20) | 93.9 | 95.7 | Menezes-Souza et al., 2014 |
| HSP83.peptide1 (Lb) | SP | Brazil | Microscopy and PCR | TL=65 (CL=45/ML=20) | 70 (HC=50/DC=20) | 63.1 | 94.3 | Menezes-Souza et al., 2014 |
| HSP83.peptide2 (Lb) | SP | Brazil | Microscopy and PCR | TL=65 (CL=45/ML=20) | 70 (HC=50/DC=20) | 63.1 | 90.0 | Menezes-Souza et al., 2014 |
| HSP83.peptide3 (Lb) | SP | Brazil | Microscopy and PCR | TL=65 (CL=45/ML=20) | 70 (HC=50/DC=20) | 89.2 | 91.4 | Menezes-Souza et al., 2014 |
| HP_LbrM.30.3350 (Lb) | RP | Brazil | Microscopy, PCR and MST | TL=43 (CL=23/ML=20) | 40 (HC=30/DC=10) | 95.4 | 85.0 | Duarte et al., 2015 |
| HP_XP_001469551.1 (Li) ^#^ | RP | Brazil | Microscopy and PCR | TL=57 (CL=27/ML=30) | 55 (HC=40/DC=15) | 100.0 | 98.2 | Carvalho et al., 2017 |
| HP_XP_001566959.1 (Lb) ^#^ | RP | Brazil | Microscopy, MST and PCR | TL=45 (CL=20/ML=25) | HC=50 | 100.0 | 98.0 | Lima et al., 2017 |
| HP_XP_001566959.1 (Lb) ^#^ | RP | Brazil | Microscopy and PCR | TL=40 (CL=15/ML=25) | 143 (HC=75/DC=68) | 100.0 | 97.8 | Lima et al., 2018 |
| HP_XP_003886492.1 (Li) ^#^ | RP | Brazil | Microscopy and PCR | TL=50 (CL=25/ML=25) | 75 (HC=25/DC=50) | 100.0 | 100.0 | Lage et al. 2019 |
| T26-U2 (like - H2b) (Lp) | RP | Colombia and Peru | Serologic | TL=78 | DC=39 | 58.0 | 87.0 | Montoya et al., 1997 |
| T26-U4 (like - HSP70) (Lp) | RP | Colombia and Peru | Serologic | TL=78 | DC=39 | 76.9 | 97.0 | Montoya et al., 1997 |
| Kmp11 (Li) | RP | Brazil | MST, serologic, histopathology and/ or therapeutic test | TL=102 (CL=49/ML=53) | HC=88 | 71.7 | 53.4 | Souza et al., 2013 |
| Lb6H (Lb) ^#^ | RP | Brazil | Microscopy, PCR and/or histopathology | TL=219 | 281 (HC=68/DC=213) | 100.0 | 93.6 | Sato et al., 2017 |
| Lb8E (Lb) | RP | Brazil | Microscopy, PCR and/or histopathology | TL=219 | HC=68 | 83.3 | 83.3 | Sato et al., 2017 |
| MAPK3 (Lb) | RP | Brazil | Microscopy and PCR | TL=65 (CL=45/ML=20) | 70 (HC=50/DC=20) | 83.1 | 71.4 | Menezes-Souza et al., 2015 |
| MAPK4 (Lb) | RP | Brazil | Microscopy and PCR | TL=65 (CL=45/ML=20) | 70 (HC=50/DC=20) | 75.4 | 97.1 | Menezes-Souza et al. 2015 |
| Peroxidoxin (Lb) ^#^ | RP | Brazil | Microscopy and PCR | TL=65 (CL=45/ML=20) | 70 (HC=50/DC=20) | 98.5 | 100.00 | Menezes-Souza et al., 2014a |
| Putative IgE histamine releasing factor (Li) ^#^ | RP | Brazil | Microscopy and PCR | TL=24 (CL=12/ML=12) | 28 (HC=20/DC=8) | 100.0 | 100.0 | Coelho et al. 2016 |
| Ribosomal protein L25 (23083) (Lb) | SP | Peru | Microscopy and culture | TL=20 | 19 (HC=9/DC=10) | 30.0 | 89.5 | Gonzáles et al., 2002 |
| Ribosomal protein L25 (23085) (Lb) | SP | Peru | Microscopy and culture | TL=20 | 19 (HC=9/DC=10) | 35.0 | 89.5 | Gonzáles et al., 2002 |
| Ribosomal protein L25 (23089) (Lb) | SP | Peru | Microscopy and culture | TL=20 | 19 (HC=9/DC=10) | 40.0 | 89.5 | Gonzáles et al., 2002 |
| Small myristoylated protein-3 (Li) ^#^ | RP | Brazil | Microscopy and PCR | TL=40 (CL=15/ML=25) | 100 (HC=35/DC=65) | 100.0 | 99.0 | Salles et al., 2019 |
| Small myristoylated protein-3.peptide 1^#^ | SP | Brazil | Microscopy and PCR | TL=40 (CL=15/ML=25) | 100 (HC=35/DC=65) | 94.50 | 92.50 | Salles et al., 2019 |
| Tryparedoxin peroxidase (Lb) ^#^ | RP | Brazil | Microscopy, PCR and MST | TL=43 (CL=23/ML=20) | 40 (HC=30/DC=10) | 100.0 | 100.0 | Duarte et al., 2015 |
| β-tubulin (Lb) ^#^ | RP | Brazil | Microscopy and PCR | TL=40 (CL=15/ML=25) | 143 (HC=75/DC=68) | 100.0 | 97.8 | Lima et al., 2018 |
| β-tubulin (Lb) | RP | Brazil | Microscopy, PCR and MST | TL=43 (CL=23/ML=20) | 40 (HC=30/DC=10) | 100.0 | 82.5 | Duarte et al., 2015 |
| A10 (Lb) ^#^ | SP | Brazil | Microscopy and PCR | TL=50 (CL=20/ML=30) | DC=10 | 100.0 | 100.0 | Costa et al., 2016 |
| B7 (Lb) ^#^ | SP | Brazil | Microscopy and PCR | TL=50 (CL=20/ML=30) | DC=10 | 100.0 | 100.0 | Costa et al., 2016 |
| B10 (Lb) | SP | Brazil | Microscopy and PCR | TL=50 (CL=20/ML=30) | DC=10 | 66.0 | 100.0 | Costa et al., 2016 |
| C11 (Lb) | SP | Brazil | Microscopy and PCR | TL=50 (CL=20/ML=30) | DC=10 | 60.0 | 100.0 | Costa et al., 2016 |
| C12 (Lb) ^#^ | SP | Brazil | Microscopy and PCR | TL=50 (CL=20/ML=30) | DC=10 | 100.0 | 100.0 | Costa et al., 2016 |
| H7 (Lb) ^#^ | SP | Brazil | Microscopy and PCR | TL=50 (CL=20/ML=30) | DC=10 | 100.0 | 100.0 | Costa et al., 2016 |
| Con-A-bound fraction (Lb) | PP | Brazil | Microscopy and Serologic | TL=58 | 171 (HC=49/DC=122) | 81.0 | 58.0 | Gomes-Silva et al., 2008 |
| Con-A-bound fraction (La) | PP | Brazil | Microscopy and Serologic | TL=58 | 171 (HC=49/DC=122) | 19.0 | 71.0 | Gomes-Silva et al., 2008 |
| Jaca-bound fraction (Lb) | PP | Brazil | Microscopy and Serologic | TL=58 | 171 (HC=49/DC=122) | 60.0 | 66.0 | Gomes-Silva et al., 2008 |
| Jaca-bound fraction (La) | PP | Brazil | Microscopy and Serologic | TL=58 | 171 (HC=49/DC=122) | 38.0 | 65.0 | Gomes-Silva et al., 2008 |

HP – hypothetical protein; HC – healthy control; DC – disease control; RP –recombinant protein; PP – purified protein; SP – synthetic peptide; La – *L. amazonensis*; Lb – *L. braziliensis;* Li – *L. infantum;* Lp – *L. peruviana;* ^#^ – antigenic target presenting sensitivity and specificity above 90%
