# Supplementary material for: Potential antigenic targets used in immunological tests for diagnosis of tegumentary leishmaniasis: A systematic review
Source: PLoS One. 2021 May 27;16(5):e0251956. doi: 10.1371/journal.pone.0251956 (PMC8158869; doi:10.1371/journal.pone.0251956)
Supplement: S3 Table — (DOCX) [file pone.0251956.s004.docx]

**S3 Table.** Antigenic targets used in ELISA for diagnosis of cutaneous leishmaniasis.

| **Antigen *(Leishmania* species)** | **Antigen Type** | **Origin of samples** | **Reference standard test** | **CL patients** | **Control (total)** | **Sensitivity (%)** | **Specificity (%)** | **Reference** |
| --- | --- | --- | --- | --- | --- | --- | --- | --- |
| Gp63 (Ld) | PP | Sudan | Microscopy or histopathology | 33 | 56 (HC=20/DC=36) | 52.0 | 80.4 | Jensen et al., 1996 |
| Gp63 (Lm) | PP | Sudan | Microscopy or histopathology | 33 | 56 (HC=20/DC=36) | 39.0 | 83.9 | Jensen et al., 1996 |
| GPB (Lm) | SP | Sudan | Microscopy or histopathology | 33 | 115 (HC=36/DC=79) | 82.0 | 94.8 | Jensen et al., 1996 |
| H1 (Lb) | RP | Peru | Microscopy and culture | 24 | 44 (HC=12/DC=32) | 66.6 | 90.9 | Carmelo et al., 2002 |
|  |  |  |  |  |  |  |  |  |
| H1_peptide 23061 (Lb) | SP | Peru | Microscopy and culture | 24 | 44 (HC=12/DC=32) | 12.5 | 100.0 | Carmelo et al., 2002 |
| H1_peptide 23063 (Lb) | SP | Peru | Microscopy and culture | 24 | 44 (HC=12/DC=32) | 0.0 | 100.0 | Carmelo et al., 2002 |
| H1_peptide 23065 (Lb) | SP | Peru | Microscopy and culture | 24 | 44 (HC=12/DC=32) | 11.7 | 100.0 | Carmelo et al., 2002 |
| H1_peptide 23067 (Lb) | SP | Peru | Microscopy and culture | 24 | 44 (HC=12/DC=32) | 46.8 | 90.9 | Carmelo et al., 2002 |
| H1_peptide 23069 (Lb) | SP | Peru | Microscopy and culture | 24 | 44 (HC=12/DC=32) | 18.8 | 100.0 | Carmelo et al., 2002 |
| H1_peptide 23071 (Lb) | SP | Peru | Microscopy and culture | 24 | 44 (HC=12/DC=32) | 12.9 | 97.7 | Carmelo et al., 2002 |
| H1_peptide 23073 (Lb) | SP | Peru | Microscopy and culture | 24 | 44 (HC=12/DC=32) | 0.0 | 100.0 | Carmelo et al., 2002 |
| H2A (Li) | RP | Brazil | MST, serologic, histopathology and/ or therapeutic test | 49 | DC=92 | 69.8 | 67.0 | Souza et al., 2013 |
| H2B (Li) | RP | Brazil | MST, serologic, histopathology and/ or therapeutic test | 49 | HC=88 | 77.8 | 61.3 | Souza et al., 2013 |
| H3 (Li) | RP | Brazil | MST, serologic, histopathology and/ or therapeutic test | 49 | HC=88 | 50.6 | 55.6 | Souza et al., 2013 |
| H4 (Li) | RP | Brazil | MST, serologic, histopathology and/ or therapeutic test | 49 | HC=88 | 60.4 | 54.5 | Souza et al., 2013 |
| HSP70 (Li) | RP | Brazil | MST, serologic, histopathology and/ or therapeutic test | 49 | DC=92 | 64.1 | 86.9 | Souza et al., 2013 |
| HSP70 (Lb) | RP | Peru | Culture | 30 | 36 (HC=20/DC=16) | 83.3 | 91.7 | Zurita et al., 2003 |
| HSP70 (1–114) (Lb) | RP | Peru | Culture | 30 | 36 (HC=20/DC=16) | 6.6 | 100.0 | Zurita et al., 2003 |
| HSP70 (109–245) (Lb) | RP | Peru | Culture | 30 | 36 (HC=20/DC=16) | 3.3 | 100.0 | Zurita et al., 2003 |
| HSP70 (240–357) (Lb) | RP | Peru | Culture | 30 | 36 (HC=20/DC=16) | 0.0 | 100.0 | Zurita et al., 2003 |
| HSP70 (352–518) (Lb) | RP | Peru | Culture | 30 | 36 (HC=20/DC=16) | 30.0 | 97.2 | Zurita et al., 2003 |
| HSP70 (513–663) (Lb) | RP | Peru | Culture | 30 | 36 (HC=20/DC=16) | 73.3 | 100.0 | Zurita et al., 2003 |
| HSP83 (Li)^#^ | RP | Brazil | MST and/or histopathology and IFAT | 12 | 20 (HC=10/DC=10) | 100.0 | 100.0 | Celeste et al., 2004 |
| HSP83 (Li) ^#^ | RP | Brazil | Microscopy and Immunological | 12 | DC=79 | 100.0 | 97.5 | Celeste et al., 2014 |
| HSP83.1 (Lb) ^#^ | RP | Brazil | Microscopy and PCR | 45 | 70 (HC=50/DC=20) | 95.6 | 95.7 | Menezes-Souza et al., 2014b |
| HSP83.peptide1 (Lb) | SP | Brazil | Microscopy and PCR | 45 | 70 (HC=50/DC=20) | 71.1 | 94.3 | Menezes-Souza et al., 2014b |
| HSP83.peptide2 (Lb) | SP | Brazil | Microscopy and PCR | 45 | 70 (HC=50/DC=20) | 64.4 | 90.0 | Menezes-Souza et al., 2014b |
| HSP83.peptide3 (Lb) ^#^ | SP | Brazil | Microscopy and PCR | 45 | 70 (HC=50/DC=20) | 95.6 | 91.4 | Menezes-Souza et al., 2014b |
| HP_XP_001566959.1) (Lb) ^#^ | RP | Brazil | Microscopy, IDRM and PCR | 20 | HC=50 | 100.0 | 98.0 | Lima et al., 2017 |
| Kmp11 (Li) | RP | Brazil | MST, serologic, histopathology and/ or therapeutic test | 49 | HC=88 | 55.7 | 53.4 | Souza et al., 2013 |
| Lbk39 (Lb) | RP | Brazil | Microscopy | 74 | 63 (HC=50/DC=13) | 88.0 | 98.0 | Souza et al., 2019 |
| NGP 0204 | PP | Brazil | Microscopy and culture | 30 | 119 (HC=60/DC=59) | 70.0 | 60.5 | Souza et al., 2018 |
| NGP 2203 | PP | Brazil | Microscopy and culture | 30 | 119 (HC=60/DC=59) | 93.3 | 51.3 | Souza et al., 2018 |
| NGP 2333 | PP | Brazil | Microscopy and culture | 30 | 119 (HC=60/DC=59) | 93.3 | 64.7 | Souza et al., 2018 |
| NGP 2334 | PP | Brazil | Microscopy and culture | 30 | 119 (HC=60/DC=59) | 90.0 | 58.8 | Souza et al., 2018 |
| Peroxidoxin (Lb) ^#^ | *RP* | Brazil | Microscopy and PCR | 45 | 70 (HC=50/DC=20) | 100.0 | 100.0 | Menezes-Souza et al., 2014a |
| Superoxide Dismutase (Lm) | RP | Iran | Microscopy | 30 | 41 (HC=20/DC=21) | 53.6 | 97.6 | Yeganeg et al., 2009 |
| Superoxide dismutase (Lp) | PP | Peru | Micorscopy | 68 | 32 (HC=12/DC=20) | 88.2 | 33.3 | Marin et al., 2009 |
| iron–superoxide dismutase (La) | PP | Colombia | Microscopy | 51 | HC=10 | 82.4 | 100.0 | Longoni et al., 2014 |
| iron–superoxide dismutase (Lp) | PP | Colombia | Microscopy | 51 | HC=10 | 11.7 | 100.0 | Longoni et al., 2014 |
| P1 _ like Gp63 (Lb) | SP | Brazil | ELISA | 57 | DC=30 | 72.0 | 86.6 | Link et al ,2017 |
| MIX (P1 + P2 + P3) _ like Gp63 (Lb) | SP | Brazil | ELISA | 57 | DC=30 | 79.0 | 90.0 | Link et al ,2017 |
| 36 kDa e 48–56 kDa (Lb) | PP | Brazil | Microscopy | 60 | 177 (HC=66/DC=111) | 93.3 | 68.9 | Skraba et al., 2014 |
| Fraction 8 - Peak 2 (Lb) | PP | Brazil | Microscopy, MST and IFAT | 48 | 114 (HC=48/DC=66) | 85.4 | 91.2 | Vidgal et al., 2008 |

HC - healthy control; DC - disease control; RP - recombinant protein; PP - purified protein; SP - synthetic peptide; ^#^ - antigenic target presenting sensitivity and specificity above 90%.
