# Supplementary material for: Potential antigenic targets used in immunological tests for diagnosis of tegumentary leishmaniasis: A systematic review
Source: PLoS One. 2021 May 27;16(5):e0251956. doi: 10.1371/journal.pone.0251956 (PMC8158869; doi:10.1371/journal.pone.0251956)
Supplement: S4 Table — (DOCX) [file pone.0251956.s005.docx]

**S4 Table.** Antigenic targets used for diagnosis of cutaneous leishmaniasis by other tests.

| **Antigen *(Leishmania* species)** | **Test format evaluated** | **Origin of samples** | **Reference standard test** | **CL patients** | **Control (total)** | **Sensitivity (%)** | **Specificity (%)** | **Reference** |
| --- | --- | --- | --- | --- | --- | --- | --- | --- |
| Peroxidoxin (pAb and mAb) | ICT | Suriname | Microscopy | 79 | DC=14 | 36.7 | 85.7 | Schallig et al., 2019 |
| Peroxidoxin (pAb and mAb) | ICT | Afghanistan | Microscopy and/or PCR | 257 | DC=17 | 65.4 | 100.0 | Vink et al., 2018 |
| Peroxidoxin (pAb and mAb) | ICT | Morocco | Microscopy and/or PCR | 136 | DC=83 | 67.6 | 94.0 | Bennis et al., 2018 |
| Peroxidoxin (pAb and mAb) | ICT | Sri Lanka | PCR | 59 | HC=22 | 35.6 | 100.0 | de Silva et al., 2017 |
| IS2-2B4 - A11 (Lt) and XLVI-5B8- B3 (Lm) (mAb)^#^ | IHC | Iran | Cytology or histopathology and PCR | 100 | DC=30 | 96.0 | 100.0 | Shirian et al., 2014 |
| G2D10 (Lg) (mAb) | IHC | Central America | Culture | 41 | DC=20 | 51.2 | 100.0 | Kenner et al., 1999 |

HC - healthy control; DC - disease control; mAb - monoclonal antibody; pAb - polyclonal antibody; Lt - *Leishmania tropica*; Lm - *Leishmania major*; Lg - *Leishmania gerbilli*; ^#^ - antigenic target presenting sensitivity and specificity above 90%.
