# Supplementary material for: Potential antigenic targets used in immunological tests for diagnosis of tegumentary leishmaniasis: A systematic review
Source: PLoS One. 2021 May 27;16(5):e0251956. doi: 10.1371/journal.pone.0251956 (PMC8158869; doi:10.1371/journal.pone.0251956)
Supplement: S5 Table — (DOCX) [file pone.0251956.s006.docx]

**S5 Table.** Antigenic targets used in ELISA for diagnosis of mucosal leishmaniasis.

| **Antigen *(Leishmania* species)** | **Antigen Type** | **Origin of samples** | **Reference standard test** | **ML patients** | **Control (total)** | **Sensitivity (%)** | **Specificity (%)** | **Reference** |
| --- | --- | --- | --- | --- | --- | --- | --- | --- |
| Acidic ribosomal protein family_LiP2aQ (*Li*) | RP | Colombia / Peru | Microscopy and IFAT | 21 | DC=30 | 76.0 | 100.0 | Soto et al., 1996 |
| Acidic ribosomal protein family_LiP2bQ (*Li*) | RP | Colombia/Peru | Microscopy and IFAT | 21 | DC=30 | 42.0 | 100.0 | Soto et al., 1996 |
| H2A (Li) | RP | Brazil | MST, serologic, histopathology and/ or therapeutic test | 53 | DC=92 | 56.3 | 79.1 | Souza et al., 2013 |
| H2B (*Li*) | RP | Brazil | MST, serologic, histopathology and/ or therapeutic test | 53 | HC=88 | 86.0 | 61.3 | Souza et al., 2013 |
| H3 (*Li*) | RP | Brazil | MST, serological, histopathology and/ or therapeutic test | 53 | HC=88 | 42.9 | 69.3 | Souza et al., 2013 |
| H4 (*Li*) | RP | Brazil | MST, serologic, histopathology and/ or therapeutic test | 53 | HC=88 | 80.3 | 54.5 | Souza et al., 2013 |
| HSP70 (*Li*) | RP | Brazil | MST, serologic, histopathology and/ or therapeutic test | 53 | DC=92 | 79.2 | 92.3 | Souza et al., 2013 |
| HSP70 (Lb) | RP | Peru | Culture | 20 | 36 (HC=20/DC=16) | 85.0 | 91.7 | Zurita et al., 2003 |
| HSP70 (1–114) (Lb) | RP | Peru | Culture | 20 | 36 (HC=20/DC=16) | 25.0 | 100.0 | Zurita et al., 2003 |
| HSP70 (109–245) (Lb) | RP | Peru | Culture | 20 | 36 (HC=20/DC=16) | 30.0 | 100.0 | Zurita et al., 2003 |
| HSP70 (240–357) (Lb) | RP | Peru | Culture | 20 | 36 (HC=20/DC=16) | 25.0 | 100.0 | Zurita et al., 2003 |
| HSP70 (352–518) (Lb) | RP | Peru | Culture | 20 | 36 (HC=20/DC=16) | 50.0 | 97.2 | Zurita et al., 2003 |
| HSP70 (513–663) (Lb) | RP | Peru | Culture | 20 | 36 (HC=20/DC=16) | 65.0 | 100.0 | Zurita et al., 2003 |
| HSP83 (*Li*) ^#^ | RP | Brazil | MST and/or histopathology and IFAT | 14 | 20 (HC=10/DC=10) | 100.0 | 100.0 | Celeste et al., 2004 |
| HSP83 (*Li*) ^#^ | RP | Brazil | Microscopy and Immunological | 14 | DC=79 | 100.0 | 97.5 | Celeste et al., 2014 |
| HSP83.1 (*Lb*) ^#^ | RP | Brazil | Microscopy and PCR | 20 | 70 (HC=50/DC=20) | 90.0 | 95.7 | Menezes-Souzaet al., 2014b |
| HSP83.peptide1 (*Lb*) | SP | Brazil | Microscopy and PCR | 20 | 70 (HC=50/DC=20) | 55.0 | 94.3 | Menezes-Souzaet al., 2014b |
| HSP83.peptide2 (*Lb*) | SP | Brazil | Microscopy and PCR | 20 | 70 (HC=50/DC=20) | 50.0 | 90.0 | Menezes-Souzaet al., 2014b |
| HSP83.peptide3 (*Lb*) | SP | Brazil | Microscopy and PCR | 20 | 70 (HC=50/DC=20) | 75.0 | 91.4 | Menezes-Souzaet al., 2014b |
| Kmp11 | RP | Brazil | MST, serologic, histopathology and/ or therapeutic test | 53 | HC=88 | 74.0 | 71.5 | Souza et al., 2013 |
| HP_ XP_001566959.1 (Lb) ^#^ | RP | Brazil | Microscopy, MST and PCR | 25 | HC=50 | 100.0 | 98.0 | Lima et al., 2017 |
| HP_XP_001467126.1 (L.i) ^#^ | RP | Brazil | Microscopy and PCR | 23 | 58 (HC=35/DC=23) | 100.0 | 100.0 | Dias et al., 2018 |
| Peroxidoxin (*Lb*) ^#^ | RP | Brazil | Microscopy and PCR | 20 | 70 (HC=50/DC=20) | 95.0 | 100.00 | Menezes-Souza et al., 2014a |
| Iron-Superoxide dismutase (Lb) | PP | Peru | Microscopy | 45 | 32 (HC=12/DC=20) | 93.3 | 32.4 | Marin et al., 2009 |

HC - Healthy control; DC - Disease control; RP - Recombinant protein; PP - Purified protein; SP - synthetic peptide; ^#^ - antigenic target presenting sensitivity and specificity above 90%
